# Building Tobacco Treatment Capacity and Sustainability: A Qualitative Study on Implementation of Community Pharmacy Technicians as Community Health Workers

**DOI:** 10.3390/pharmacy14050114

**Published:** 2026-08-03

**Authors:** Cindy V. Valencia, Robin L. Corelli, Shin-Ping Tu, Melissa M. Gosdin, Elisa K. Tong

**Affiliations:** 1 Center for Healthcare Policy and Research, University of California Davis, Sacramento, CA 95817, USA; cvvalencia@health.ucdavis.edu (C.V.V.); mmgosdin@health.ucdavis.edu (M.M.G.); 2 Tobacco Cessation Policy Research Center, University of California Davis Comprehensive Cancer Center, Sacramento, CA 95817, USA; robin.corelli@ucsf.edu (R.L.C.); sptu@health.ucdavis.edu (S.-P.T.); 3 Department of Clinical Pharmacy, University of California San Francisco, San Francisco, CA 94143, USA; 4 Department of Internal Medicine, University of California Davis, Sacramento, CA 95817, USA

**Keywords:** tobacco cessation, smoking cessation, implementation science, community pharmacy, qualitative research, community health workers

## Abstract

Community pharmacies are increasingly providing health care services like tobacco treatment and disease-related management but need sustainable models for implementation. As several state Medicaid programs reimburse for community health worker (CHW) services, one promising model is to have pharmacy technicians engage with patients as designated CHWs. This qualitative study interviewed staff in seven California pharmacies about their experience with and perspectives on implementing community pharmacy technicians as CHWs. Guided by implementation science frameworks, data were hand-coded and analyzed iteratively using thematic analysis. Relative advantage and compatibility of CHWs was high across all pharmacies. Pharmacy culture aligned with CHW values, and CHW integration helped formalize the work that pharmacy staff were already doing. Challenges included the complexity of billing and obtaining payment for services provided. Despite these barriers, pharmacies successfully integrated and expanded their CHW services, ranging from social service connections to tobacco treatment or disease-related management education. Pharmacy peer networks, health plan champions and patient outcome improvements accelerated diffusion. Community pharmacies provide a trusted setting for delivering both CHW education and extending health care services, but broad implementation will need health plans to improve policy and procedure with guidance specifically for the pharmacy context that helps overcome administrative barriers.

## 1. Introduction

Community pharmacies are highly accessible health care locations in the United States [1] and increasingly provide services such as tobacco treatment and chronic disease prevention and management [2], but sustainable models are needed to support this expanding scope of care. Despite the availability of effective, evidence-based interventions, tobacco use remains disproportionately high among socioeconomically disadvantaged populations, including individuals on Medicaid (Medi-Cal in California) [3]. Medi-Cal members often struggle with structural barriers to care, along with social needs (e.g., food, transportation, and housing). Increasing access to evidence-based tobacco treatment while addressing social drivers of health (SDOH) in community pharmacy settings may help decrease tobacco-related disparities among Medi-Cal populations.

Expanding tobacco treatment into community pharmacies is an effective strategy to help patients quit [4,5,6]. Pharmacies have frequent patient contact, extended service hours, and walk-in access, particularly in rural and underserved communities [1]. A recent qualitative study of community pharmacies found that while tobacco treatment services were perceived as compatible with pharmacy workflows, the complexity of billing and reimbursement remains a key barrier to sustainability [7]. In California, pharmacists are now authorized to furnish all FDA-approved tobacco cessation medications, including non-nicotine therapies such as varenicline and bupropion [8], expanding the role of community pharmacies as viable sites for tobacco treatment. However, operational and reimbursement models to sustain these services in community pharmacies are limited.

Integrating community health workers (CHWs) into community pharmacies is a novel approach to address SDOH, along with treating tobacco use dependence. CHWs are trusted frontline public health workers whose roles include SDOH screening, health education, care navigation, and supportive services, all of which are reimbursable under the new Medi-Cal CHW benefit [9]. Many CHWs’ roles align with those of pharmacy technicians [10,11,12], who often come from the communities they serve [13,14]. Studies on pharmacy technicians trained as CHWs show improved pharmacy services, increased referrals for social needs, and reduced out-of-pocket prescription costs [15,16,17,18].

While tobacco treatment integration in community pharmacies has been explored [7], greater understanding is needed of the use of CHW-trained pharmacy technicians to support patients in the treatment of nicotine dependence. Seven pharmacies that had previously trained pharmacy technicians and clerks as CHWs expanded this model to include tobacco treatment. With Medi-Cal reimbursement available for CHW services, this approach offers a sustainable funding model. This qualitative study explores the barriers and facilitators of integrating CHW services by pharmacy technicians into community pharmacy settings to support tobacco treatment.

## 2. Materials and Methods

### 2.1. Study Design

A qualitative study was conducted through semi-structured interviews with community pharmacies integrating CHW services provided by pharmacy technicians to support tobacco treatment. This study is reported based on recommendations in the Consolidated Criteria for Reporting Qualitative Research (COREQ) checklist [19].

### 2.2. Qualitative Interview Approach and Theoretical Framework

Semi-structured interviews were conducted with each participant. The Consolidated Framework for Implementation Research (CFIR) [20] was applied to develop an interview guide for identifying barriers and facilitators related to intervention characteristics, workflow integration, staffing, reimbursement, and organizational readiness. The guiding questions that informed the interview script are presented in Table 1. The CFIR informed the development of the semi-structured interview guide by identifying implementation determinants.

Rogers’ Diffusion of Innovations Theory was applied as a guiding framework [21], encompassing five main factors that influence the successful adoption of CHW services: (a) relative advantage (e.g., the degree to which the new service is perceived by pharmacy staff as better than previous practices); (b) compatibility (e.g., the new service is perceived as being consistent with the values, past experiences, and needs of the community pharmacy setting); (c) complexity (e.g., the degree which the new service is perceived as difficult to implement and use); (d) trialability (e.g., the degree to which experimentation is possible with the new service); and (e) observability (e.g., the ability to see the impact of implementing the new service). Rogers’ Diffusion of Innovations Theory constructs provided the analytic lens to understand how pharmacy teams perceived, adopted and normalized CHW integration. Rogers’ Diffusion of Innovations Theory constructs are incorporated in the CFIR and, in contrast to the expansive domains and constructs of the CFIR, provided the focused lens to understand intervenable barriers and facilitators.

### 2.3. Study Site Selection, Recruitment and Eligibility Criteria

Study sites included pharmacies in California affiliated with the Community Pharmacy Enhanced Services Network (CPESN), a clinically integrated organization of community pharmacies providing medication optimization and enhanced patient care services in 47 states and the District of Columbia [22]. After consultation with the lead CPESN administrator in California, seven independently owned pharmacies were identified as potential study sites. Three pharmacies were active participants in a CPESN-led tobacco treatment initiative, three pharmacies were participants in a Medi-Cal CHW capacity-building pilot, and one pharmacy was identified through the CPESN.

Pharmacy owners at these locations received an email invitation from a researcher at the University of California, Davis (Sacramento, CA, USA). Participants were informed of the purpose of the study, the interviewer’s role on the research team, and the study objectives before participation. To be eligible, pharmacies were required to meet the following criteria: (a) a pharmacist or staff member (technician/clerk) had completed a CHW training program, (b) had experience planning for CHW service integration, or (c) had experience implementing a CHW program in the pharmacy setting. CHW training included either the University of Southern California CHW Certificate Program or the CEimpact Pharmacy-Based CHW Training. California CHW Medi-Cal policy defines CHW services, which may include health education, social needs screening, care coordination, and resource navigation, with reimbursement available when supervised by an eligible provider, including pharmacists. All seven pharmacies met the minimum qualifications and agreed to participate in the structured interviews.

### 2.4. Data Collection and Analysis

Interviews, designed to be 30 min in duration, were conducted via the Zoom platform, with audio recordings being subsequently de-identified and transcribed by a professional service. The participants received a $50 Amazon.com gift card as compensation for their time.

One investigator, C.V.V. (female, PhD), a public health researcher, conducted the interviews, and one investigator, M.M.G. (female, PhD), a qualitative researcher, performed the qualitative thematic analysis, using Braun and Clarke’s reflexive thematic analysis approach [23]. Analysis was conducted manually by M.M.G., who has extensive experience in health services research. M.M.G. independently engaged in analysis by reading and re-reading to preserve context and listened to the interview recordings when clarification was needed. M.M.G. created a codebook deductively based on CFIR domains, and Rogers’ Diffusion of Innovation Theory Codes were applied to the first two transcripts and then were modified and applied to the last four transcripts.

Strategies to enhance the rigor of this study included iterative refinement of codes using constant comparison analysis within and across transcripts. Final codes were then collapsed into categories based on commonalities, and themes were created. M.M.G. and C.V.V. met regularly to discuss and debrief about the data and emergent patterns. All interviews were conducted by C.V.V., who was familiar with the data and able to answer questions as they came up. S.P.T. participated in debriefing sessions and was available to resolve discrepancies. C.V.V., M.M.G. and S.P.T. had no prior professional experience collaborating with community pharmacies.

### 2.5. Ethical Considerations

This study was approved by the University of California, Davis. The IRB study protocol number is 2020762-4, and this study is not considered human subjects research.

## 3. Results

### 3.1. Interview Participants and Pharmacy Characteristics

A subset of pharmacy personnel (n = 13) representing seven pharmacies were recruited, including six pharmacists (four owners and two managers) and seven pharmacy technicians. Each participant completed one individual interview, for a total of 13 interviews. The participating pharmacies represented geographically and operationally diverse settings in California (Table 2). Although some pharmacies were located in urban areas, one was located in a rural area. Pharmacies commonly provided prescription dispensing, immunizations, medication adherence support and patient counseling services. CHW training had been undertaken at the University of Southern California or as part of another program (CEimpact).

### 3.2. Findings

Findings are presented as implementation determinants influencing adoption. Table 3 maps the CFIR domains to Rogers’ Diffusion of Innovation constructs and findings. Themes and select representative quotes are presented for Rogers’ Diffusion of Innovation constructs in Table 4. Table 4 includes themes related to five constructs: compatibility, relative advantage, complexity, trialability, and observability.

#### 3.2.1. Compatibility Construct: Fit Within Existing Pharmacy Workflows, Values and Cultures

Extension of CHW services. Respondents emphasized that compatibility was the strongest when CHW tasks were integrated into routine pharmacy touchpoints, such as vaccine appointments, long-acting injectable visits, prescription pick-ups, blood pressure monitor purchases, and medication therapy management reviews.

Alignment with pharmacy mission. Participants widely agreed that CHW work aligns naturally with the community pharmacy identity including education, coaching, relationship building, and helping people navigate health challenges. Community pharmacies share a desire to be impactful, trusted and connected to the communities they serve. Collaborative team norms among pharmacists, technicians, and clerks further support the CHW model, while technicians’ pride in their work and pharmacists’ sense of purpose provide additional motivation for engagement.

#### 3.2.2. Relative Advantage Construct: Improvement over Standard Practice

Enhanced patient trust and engagement. Across all sites, participants described CHW integration as expanding the pharmacy’s value for both patients and the business.

Addressing social drivers of health. Respondents framed CHW work as a way to address patients’ social needs that pharmacy personnel were already identifying but previously lacked the standardized workflows or time to act on.

Reimbursement to support expansion. The Medi-Cal CHW benefit provides a formal reimbursement pathway to sustain and expand CHW services in pharmacies.

#### 3.2.3. Complexity Construct: Barriers to the CHW Model

Health plan process and forms. Participants reported difficulty understanding the requirements for becoming a credentialed provider. Further, several respondents reported credentialing delays (e.g., 3–6 months in many cases).

Pharmacy reimbursement. While participants found the clinical work manageable, billing was consistently identified as the major barrier. Several respondents noted unclear or inconsistent billing requirements, uncertainty about which CHW services are reimbursable, and documentation burden as key challenges. Working with multiple payers further complicated workflows, often requiring the use of multiple systems to fulfill required documentation for CHW reimbursement (e.g., office support and CMS forms).

#### 3.2.4. Trialability Construct: Pilots to Experiment with CHW Workflow

Health plan champions. Several interviewees relied on small pilots to experiment with CHW workflows. A designated health plan contact was identified as critical to navigating the administrative learning process and to sustaining CHW integration. One health plan pharmacy leader designed and implemented a small capacity-building pilot to help cover the initial CHW training costs using programs such as the University of Southern California CHW Certificate Program and CE Impact Pharmacy-Based CHW training. Health plan support also reduced uncertainty through documentation templates and investments in the model.

Starting with one service. Participants shared that CHW integration was incremental, typically beginning with one service, such as blood pressure checks, tobacco cessation, or diabetes education. Offering CHW services during certain touchpoints allowed staff to test scripts, forms, and documentation methods before expanding.

Iterative learning. Most pharmacies refined their billing processes through “trial and error” with incremental implementation, building staff confidence and reducing perceived risk. At the time of interviews, CHW integration remained in the early stages, with only one pharmacy having more than one year of implementation experience.

#### 3.2.5. Observability Construct: Visible Results of CHW Integration

Patients assisted. Interviewees described a range of CHW services offered, such as food delivery enrollment, clinic access, and transportation assistance. Respondents reported anecdotal improvements in blood pressure and medication adherence.

Peer pharmacy influence. Reimbursement success stories from peer pharmacies were consistently cited as a strong motivator. Seeing peers successfully submit and receive CHW payments normalized the model and accelerated adoption. Peer communication occurred via phone, text message, and email, with one pharmacy also noting the value of participating in a local county-level CHW coalition early in the implementation process.

## 4. Discussion

This qualitative study examined barriers to and facilitators for integrating CHW services within community pharmacy settings to support tobacco treatment. Using Rogers’ Diffusion of Innovation framework, CHW integration was perceived as highly compatible with community pharmacy practice but was challenged by complexity, particularly in credentialing, billing, and reimbursement requirements.

Relative advantage and compatibility were the primary facilitators of adoption. Pharmacy culture aligned closely with CHW values, and CHW integration formalized work that pharmacy technicians, clerks, and pharmacists were already doing. Community pharmacy culture already supports deep patient relationships, coaching, identifying barriers to care, and helping vulnerable populations navigate health and social service systems. This alignment facilitated workflow integration, enhanced patient engagement and expanded service delivery. Of note, these observed benefits may reflect the formalization of patient support activities that pharmacies were already doing rather than accepting a new care delivery model. Pharmacy technicians and clerks are well-positioned to support tobacco cessation through routine patient interactions and existing workflow integration [24,25]. CHW training may augment this role by providing additional skills in social needs assessment, care coordination, resource navigation, and follow-up, thereby addressing barriers to successful quit attempts. Similar findings have been reported in prior implementation studies demonstrating that pharmacy innovations are adopted when integrated into existing workflows and aligned with professional roles [26,27,28,29]. Additionally, we found results similar to prior tobacco cessation and CHW [30,31] studies demonstrating that patient-centered support models improve engagement and facilitate preventive care delivery [24,25,30,31].

Although CHW service delivery was viewed as feasible, implementation was challenged by administrative complexity. Credentialing delays, billing uncertainty, documentation burden, and variable payer requirements related to Medi-Cal-managed care plans were the primary barriers to adoption and sustainability. These findings suggest that policy alone is insufficient to ensure implementation and that more detailed policy and procedure by each health plan that is specific to the pharmacy context is needed instead of broad guidance [32]. In California, where pharmacies are explicitly authorized to supervise and bill for CHW services, participants still described reimbursement processes as one of the most significant barriers. Additionally, billing guidance, pharmacy-specific implementation tools, technical assistance, and health plan champions may be necessary to support broader adoption in community pharmacy settings [28,29,33,34].

Despite these barriers, pharmacies successfully integrated CHW services using an iterative approach. Trialability facilitated implementation, with pharmacies often starting with a single patient care service, such as tobacco cessation, vaccine assessment, or blood pressure monitoring, before expanding to broader SDOH navigation. This approach reduced perceived risk and increased organizational confidence. Similar iterative and phased approaches are reported in prior community pharmacy innovation studies [26,29]. Observable benefits, including improved patient engagement, enhanced access to social services, provision of language services reflecting needs of the local community, successful patient assistance, and early reimbursement experiences, reinforced motivation to continue implementation. Peer pharmacy success stories from early adopters and health plan champions further accelerated diffusion. Visible patient outcomes can strengthen diffusion and adoption, as noted in CHW-led tobacco cessation and pharmacy innovation studies [27,31].

Community pharmacies represent a viable setting for CHW-supported tobacco cessation services. CHW training can expand the role of pharmacy technicians by strengthening their ability to address social needs, coordinate care, navigate resources, and provide follow-up support. CHWs are a heterogenous group that operates in various settings. Pharmacy technicians are strategically positioned to serve as CHWs, as they often come from the community they serve, already have frequent touchpoints with patients and possess the foundational knowledge of medications, chronic disease management and pharmacy workflow. Additionally, this model might enable community pharmacy personnel to provide longitudinal, team-based tobacco treatment that addresses both nicotine dependence and social drivers of health that influence quit attempts. However, scalability will depend on simplifying reimbursement pathways, clarifying billable services, supporting technician training, and demonstrating sustainable clinical and financial outcomes.

Strengths of this study include the in-depth qualitative approach with pharmacists and pharmacy technicians, as well as the application of Rogers’ Diffusion of Innovation framework. However, participants represented only seven independently owned pharmacies affiliated with a national network in California, limiting generalizability. First, these participating pharmacies may represent early adopters, who are highly motivated through a health plan’s pilot resources. This may overestimate feasibility and implementation success. Second, only pharmacies implementing or planning on implementing CHW models are represented, thus limiting our understanding of various implementation perspectives. Despite these limitations, the findings provide useful information regarding the implementation of CHW models in community pharmacy settings and may inform future efforts to expand tobacco treatment services.

## 5. Conclusions

Integrating pharmacy technicians as CHWs is a promising yet underdeveloped strategy to expand tobacco treatment delivery in community pharmacy settings. Although CHW services were highly compatible with community pharmacy practice, implementation was challenged by credentialing, billing, and reimbursement requirements. Broad implementation will need health plans to improve policy and procedure with guidance specifically for the pharmacy context that helps overcome administrative barriers.

CHW models may strengthen the community pharmacy’s capacity to deliver team-based, patient-centered tobacco treatment by addressing both nicotine dependence and the social and structural barriers that impact quit attempts. Our study does not measure patient-level and financial outcomes; therefore, conclusions regarding effectiveness and sustainability are preliminary and based on participant experience and perceived reimbursement success. Further research is necessary to evaluate the long-term sustainability of pharmacy-based CHW models, including successful reimbursement, patient engagement, and tobacco treatment outcomes.

## Figures and Tables

**Table 1:** Guiding questions used for semi-structured interviews with pharmacists and pharmacy technicians.

CFIR Domains	Guiding Questions
Innovation Characteristics	What is the main purpose of your CHW program?
Characteristics of Individuals	Who are the main individuals involved?
Access to Knowledge for Information	What training and resources or systems are needed to support CHW implementation?
Inner Setting	How are the CHW services integrated into your pharmacy workflow?
Outer Setting	How well has billing for CHW services worked to fund the program?
Readiness for Implementation	What are the core resources necessary for successful implementation and ongoing operation of a CHW program?

**Table 2:** Participant and pharmacy characteristics.

Site	Total Locations	Urban Status *	Years in Business	Payor Source **			CHW Training Completed
				Medicare (%)	Medi-Cal (%)	Private (%)	
A	4	Urban	69	40	20	35	CEimpact
B	2	Urban	45	25	55	15	USC and local college
C	1	Urban	6	30	25	40	USC
D	1	Rural	6	50	20	25	CEimpact
E	1	Urban	7	45	25	35	USC
F	1	Urban	8	33	33	33	USC
G	1	Urban	50	38	22	35	USC

* Urban status defined by the United States Department of Agriculture Rural–Urban classification system based on pharmacy location. ** Years in business and payor sources based on CPESN data.

**Table 3:** Mapping of CFIR domains to Rogers’ Diffusion of Innovation constructs and findings.

CFIR Domain	Rogers’ Diffusion of Innovation Construct	Key Findings
Innovation Characteristics	Relative Advantage	CHW services expanded value and created reimbursement that formalized work already occurring within the pharmacy
Characteristics of Individuals	Compatibility	Pharmacy technicians are well positioned to serve as CHWs due to alignment with existing responsibilities
Access to Knowledge for Information	Complexity and Trialability	CHW training, documentation templates and health plan support important (e.g., pilots)
Inner Setting	Compatibility	Leverage existing pharmacy touchpoints (e.g., prescription pick-up, medication therapy management visits, and immunizations)
Outer Setting	Complexity, Trialability, and Observability	Billing, credentialing and documentation barriers; health plan champions and successful reimbursement accelerated adoption
Readiness for Implementation	Trialability and Observability	Organizational leadership, trained staff, reimbursement infrastructure, integrated workflows and peer learning networks supported implementation

**Table 4:** Themes and representative quotes for Rogers’ Diffusion of Innovation Theory constructs: compatibility, relative advantage, complexity, trialability, and observability.

Theme	Representative Quotes
Compatibility: Fit within existing pharmacy workflows, values and cultures	
Extension of services	“What we’re doing is we’re implementing a new workflow…when people come in to get vaccinations…they’ll be seen by a CHW to be screened for blood pressure and tobacco use and if there’s any other sort of need that they might identify during that conversation.” (Pharmacist) “Main focus was smoking cessation to start with and then other programs that we wanted to implement. But right now, we’re focusing a lot more on vaccine efforts for children…” (Pharmacist)
Alignment with pharmacy mission	“I think my staff are very engaged. You can see the pride that they take when they identify an opportunity and they sort of get excited about it, and they are happy to help our community in whatever they can do.” (Pharmacist) “We want to be able to assist the patient with more than just doing the prescription service.” (Pharmacist)
Relative Advantage: Improvement over standard practice	
Addressing social drivers of health is already standard practice	“I would say many staff members are already doing this work to some extent. You might as well get paid for it. You might as well be able to bill for what they’re already doing.” (Pharmacist) “We’re already seeing qualitatively, people that they would fall through the cracks otherwise, if not for this program.” (Pharmacist)
Reimbursement to support expansion	“I think we’re still just exploring exactly what the CHWs can do and what is considered a billable service. I think that’s sort of our goal for 2025 is to really understand what functions our CHWs may be already providing that become billable. And then in turn, how can we expand our offerings to, I guess, provide a broader scope of offerings for patients.” (Pharmacist) “She’s (CHW) done this job long before it had a title. And we’ve never been reimbursed for services that we’ve been doing for years. So the opportunity came up for us to give titles and be able to bill, so it was amazing.” (Pharmacist)
Complexity: Barriers to the CHW model	
Health plan process and forms	“Easiest is helping patients, easiest making a call, getting them assistance and following up on it. The hardest, billing. Finding the codes that we need to bill and taking the time to bill.” (Pharmacist) “It took us about maybe six months to become credentialed as a provider with [the health plan] and connect all the dots in terms of electronic billing to be fully online.” (Pharmacist) “So I think training. Billing, training. At least guidelines, booklet, outline, something would be helpful.” (Pharmacist)
Pharmacy reimbursement	“Right now we see kind of poor reimbursement from filling prescriptions, and this is really a good way to help supplement those poor reimbursements on medications and helps our staff to maintain employment and maintain an adequate number of staff. I think it’s a good kind of adjunct program to have as part of your pharmacy.” (Pharmacist) “[H]ow do we bill properly? What codes do we need to use? Why aren’t these claims being paid? I think we’re at a point now that we’re a year and a half or so into the process to where…the billing seems to be going a lot smoother.” (Pharmacist)
Trialability: Pilots to experiment with CHW workflow	
Health plan champions	“We’ve been working with the [plan’s] director of pharmacy. While he’s not always the person that gives us the answer, he connects us with the person who knows the answer.” (Pharmacist) “They [the health plan] helped with [funding a] …scholarship …for the CHWs to become trained.” (Pharmacist)
Starting with one service	“Main focus was smoking cessation to start with and then other programs that we wanted to implement. But right now, we’re focusing a lot more on vaccine efforts…” (Pharmacist) “I actually started working just on SDOH cases… That was the majority of what I was focusing… So that was my main goal. But then we started shifting our focus into health education because we started seeing that a lot of these patients were going to the doctor, but they weren’t understanding what they were being told.” (Technician)
Iterative learning	“Billing-wise, we’re fortunate to have a very experienced billing manager and it did take in the beginning, a bit of trial and error and a lot of communication between us and the health plan to work out the kinks in the system.” (Pharmacist) “So there’s a lot of what do we do? Do we do this, do we do that? And it’s just a lot of trial and error right now. But hopefully with more people doing it and people figuring out all the nuances of it, it could become more streamlined.” (Pharmacist)
Observability: Visible results of CHW integration	
Patients assisted	“If we didn’t have that interaction with the clinic or with the hospital, this patient literally would’ve went home with no meds and not known what to do, and probably would’ve been a readmit.” (Technician) “You can literally see the relief on patients’ faces when they get help.” (Technician) “When shelters are full and we can’t help, it’s heartbreaking—those moments stick too.” (Technician)
Peer pharmacy influence	“We do have for now a standardized CHW form that a different pharmacist was able to provide for us.” (Pharmacist) “When early pharmacies shared their forms, it made the rest of us confident.” (Pharmacist) “We saw [another pharmacy’s] forms and thought, okay, this is real.” (Pharmacist) “…I wish also in the future they have some more of guidelines or experiences with other people how they do it… Everyone’s coming up with a plan based on their situation… But if there’s some sort of training… for pharmacy owners… that would be very helpful.” (Pharmacist) “[An early adopter’s] billing manager helped us set everything up.” (Pharmacist)

## Data Availability

The original contributions presented in this study are included in the article. Further inquiries can be directed to the corresponding author.
